# Bilateral Sensorineural Hearing Loss in a Patient with Primary Ciliary Dyskinesia and Concomitant SH3TC2 Gene Mutation

**DOI:** 10.3390/jcm14113692

**Published:** 2025-05-25

**Authors:** Mirko Aldè, Umberto Ambrosetti, Raffaella Guazzo, Maria Santa Rocca, Gioia Piatti

**Affiliations:** 1 Department of Clinical Sciences and Community Health, University of Milan, 20122 Milan, Italy; umberto.ambrosetti@unimi.it; 2 Audiology Unit, Department of Specialist Surgical Sciences, Fondazione IRCCS Ca’ Granda Ospedale Maggiore Policlinico, 20122 Milan, Italy; 3 Pathology Unit, Laboratory of Electron Microscopy, Siena University Hospital, 53100 Siena, Italy; raffaella.guazzo@ao-siena.toscana.it; 4 Unit of Andrology and Reproductive Medicine, University Hospital of Padova, 35128 Padova, Italy; mariasanta.rocca@aopd.veneto.it; 5 Department of Pathophysiology and Transplantation, University of Milan and Unit of Bronchopneumology, Fondazione IRCCS Ca’ Granda Ospedale Maggiore Policlinico, Via Francesco Sforza 35, 20122 Milan, Italy; gioia.piatti@unimi.it

**Keywords:** primary ciliary dyskinesia, sensorineural hearing loss, Charcot–Marie–Tooth disease, genetic, hearing assessments

## Abstract

**Background:** Primary ciliary dyskinesia (PCD) is a rare hereditary disorder caused by defective motile cilia, predominantly affecting the respiratory system. Conductive hearing loss (CHL) due to chronic otitis media with effusion (OME) is a typical feature of PCD, particularly in childhood. However, the underlying mechanisms contributing to sensorineural hearing loss (SNHL) in patients with PCD remain unclear. **Methods**: We present the case of a 52-year-old male with a clinical diagnosis of PCD, confirmed by the presence of situs inversus, chronic respiratory symptoms, and ultrastructural ciliary defects. **Results:** Despite a history of recurrent acute otitis media (AOM), the patient developed severe bilateral SNHL, a relatively uncommon and poorly understood manifestation of PCD. Genetic testing revealed a pathogenic SH3TC2 variant, a gene classically associated with Charcot–Marie–Tooth disease type 4C (CMT4C), raising the possibility of an alternative or contributory genetic etiology for the patient’s auditory dysfunction. **Conclusions**: This case highlights the importance of comprehensive audiological and genetic evaluations in PCD patients, particularly those presenting with progressive or atypical HL. The presence of a pathogenic SH3TC2 mutation suggests a potential neuropathic component to the patient’s HL, underscoring the need for further research into the intersection between ciliary dysfunction and genetic neuropathies. Early identification and intervention are critical to optimizing auditory outcomes and quality of life in affected individuals.

## 1. Introduction

Primary ciliary dyskinesia (PCD) is a rare hereditary disorder with an estimated prevalence of 1 in 10,000–20,000 individuals [1]. It is primarily inherited in an autosomal recessive manner, though autosomal dominant and X-linked patterns exist [2]. PCD is characterized by impaired mucociliary clearance, leading to recurrent upper and lower respiratory tract infections [3]. Typical symptoms include chronic wet cough, recurrent sinusitis, chronic otitis media with effusion (OME), and recurrent acute otitis media (AOM), particularly in childhood [4]. Bronchiectasis is almost universally present in adulthood. About 50% of PCD patients exhibit situs inversus or other forms of heterotaxy due to dysfunction of embryonic nodal cilia. Additionally, infertility is common due to immotile spermatozoa or defective ciliary function in the fallopian tubes [5]. PCD is genetically heterogeneous, with mutations in over 50 genes implicated in its pathogenesis [6].

Charcot–Marie–Tooth disease (CMT) is a genetically diverse group of hereditary neuropathies that affect both sensory and motor functions. It is the most prevalent inherited disorder of the peripheral nervous system, with an estimated prevalence of 1 in 2500 individuals [7]. While CMT is most commonly inherited in an autosomal dominant pattern, X-linked and autosomal recessive forms have also been identified [8]. CMT is characterized by a slowly progressive sensorimotor neuropathy, typically beginning in childhood. Clinical manifestations vary widely, with some individuals remaining asymptomatic or experiencing only mild symptoms, while others develop significant impairments. Common features include pes cavus (high-arched feet), foot drop, gait disturbances, and spinal deformities such as scoliosis. Additionally, cranial nerve involvement is frequently observed [7,9]. To date, over 90 distinct genetic mutations have been linked to CMT, each contributing to the disease’s complex phenotypic spectrum [10]. Among its various subtypes, Charcot–Marie–Tooth disease type 4C (CMT4C) is an autosomal recessive demyelinating neuropathy associated with mutations in the SH3TC2 gene. CMT4C typically presents in early childhood and is often characterized by progressive scoliosis or kyphoscoliosis, which can range from mild to severely disabling. Cranial nerve dysfunction is a hallmark feature and may lead to a range of symptoms, including diplopia or strabismus (due to oculomotor nerve impairment), sensorineural hearing loss (SNHL), vestibular dysfunction, dysphagia, tongue atrophy, and vocal cord paresis. The severity and progression of symptoms vary among individuals, highlighting the clinical heterogeneity of the disease [11].

Hearing loss (HL) is a clinically significant yet often underrecognized manifestation of both PCD and CMT. In PCD, HL is generally conductive, resulting from chronic OME [12], while CMT4C is more frequently associated with progressive SNHL due to involvement of the cochlea or auditory nerve [13]. Delayed diagnosis and inadequate treatment of HL can significantly impair quality of life. Indeed, unaddressed HL can negatively affect communication, language development, cognitive function, social interaction, and economic well-being. Management depends on the underlying etiology and severity, with options ranging from tympanostomy tube insertion for OME, to hearing aids for mild to moderate SNHL, and cochlear implantation for profound SNHL. Prompt and accurate recognition of HL is vital to facilitate effective auditory rehabilitation and ensure better long-term outcomes [14].

We present the case of an adult PCD patient with severe bilateral SNHL, in whom genetic testing revealed a pathogenic variant in SH3TC2, a gene associated with CMT4C. This raises the question of whether the patient’s SNHL is directly linked to PCD or represents a coincidental coexisting neuropathy that affects auditory function.

## 2. Case Study

### 2.1. Patient Background

A 52-year-old man with suspected PCD was referred to the Centre for Rare Diseases at Fondazione IRCCS Ca’ Granda, Ospedale Maggiore Policlinico (Milan, Italy) following a diagnosis of situs inversus and a history of recurrent respiratory infections requiring long-term treatment. The patient was born at full term and did not experience any respiratory complications during the neonatal period. His parents were first cousins. He was a former smoker (10 cigarettes per day for 20 years). The patient’s occupational background reflects diverse roles, having previously worked as a bricklayer and currently holding a position as an ecological operator. He reported recurrent episodes of bronchitis since early childhood, though he had never been diagnosed with pneumonia. At presentation, his primary complaints included daily cough and sputum production, with no associated dyspnea. Additional comorbidities included arterial hypertension (treated with ramipril), hypothyroidism (managed with levothyroxine), and recurrent trigeminal pain attacks. The patient also had a history of infertility (attributed to severe asthenospermia and moderate teratospermia), scoliosis, and HL. Finally, he reported mild balance difficulties, especially when standing on uneven ground or in poorly lit environments, and noted an inability to walk in a straight line without visual guidance. While he did not experience falls, he frequently needed to steady himself using external objects during activities that challenged his proprioceptive control.

### 2.2. Respiratory and Nasal Assessment

Pulmonary function tests were within normal limits: Forced Expiratory Volume in 1 s (FEV1) > 80%, Forced Vital Capacity (FVC) > 80%, and FEV1/FVC ratio > 70%. High-resolution computed tomography (HR-CT) of the chest revealed bilateral cylindrical bronchiectasis predominantly at the lung bases. In terms of upper airway involvement, the patient had undergone three previous nasal polypectomies. At the time of presentation, he reported anosmia and ageusia. CT of the paranasal sinuses showed extensive inflammatory changes in the maxillary sinuses, ethmoid cells, sphenoid sinus, and frontal sinuses, consistent with chronic rhinosinusitis and nasal polyposis. The Lund–MacKay score, a widely used radiologic staging system for chronic rhinosinusitis (range: 0–24), was 24, indicating severe disease. Additionally, the Sino Nasal Outcome Test-22 score (SNOT-22), a questionnaire assessing chronic rhinosinusitis symptoms (score range: 0–110), yielded a score of 46, reflecting a moderate degree of symptoms. The patient performed daily nasal washes with saline solution and used intranasally topical steroids. Allergy testing results were negative, and no drug intolerances were reported.

**Figure 1:**
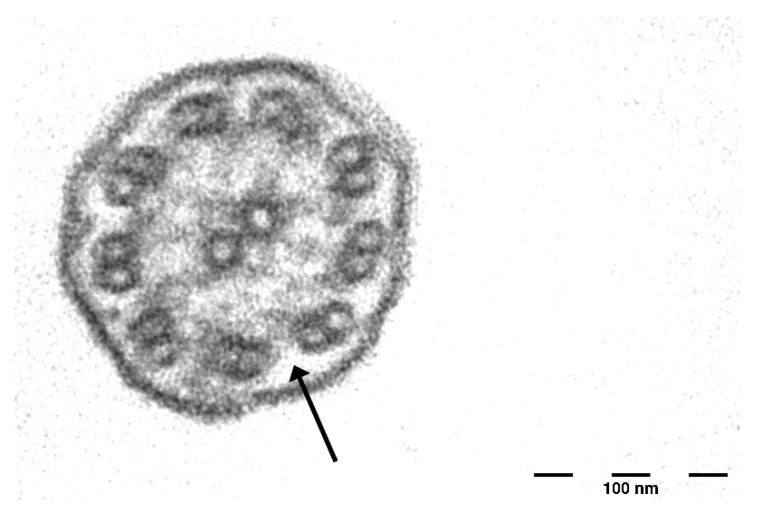
Ultrastructural analysis of respiratory cilia, demonstrating the absence of the outer dynein arm.

The saccharin test revealed pathological mucociliary transport time, with no sweet taste perception after 60 min. Ciliary motility analysis was inconclusive due to an insufficient number of ciliated cells. Transmission electron microscopy (TEM) revealed the absence of outer dynein arms in more than 50% of cilia cross-sections (Figure 1), with normal inner dynein arms, central pair, and microtubular organization.

### 2.3. Audiological and Vestibular Assessment

**Figure 2:**
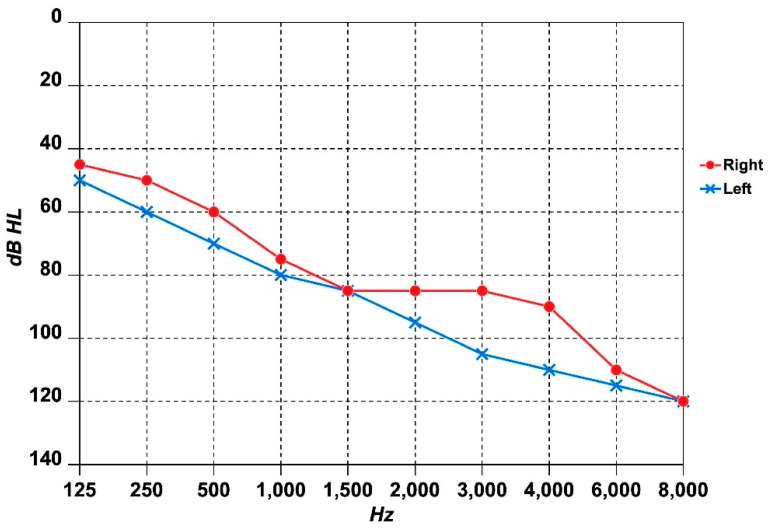
Audiogram of the patient.

During childhood the patient suffered from recurrent AOM, while he began to develop progressive bilateral SNHL in his 40 s. He has been using bilateral conventional behind-the-ear (BTE) hearing aids for the past three years. On otomicroscopic examination, the tympanic membranes appeared bilaterally intact, with no perforations or retraction pockets. However, their opaque appearance was suggestive of tympanosclerosis. Pure-tone audiometry (at frequencies from 125 Hz to 8000 Hz) revealed severe bilateral SNHL with a sloping audiometric configuration, slightly more pronounced in the left ear (Figure 2). On speech audiometry, the maximum speech intelligibility was 30% at 100 dB HL in the right ear, and 20% at 100 dB HL in the left ear. The tympanogram was type A bilaterally, while the stapedial reflexes were absent. Auditory brainstem responses (ABRs) and slow vertex responses (SVRs) confirmed the hearing thresholds, while otoacoustic emissions (OAEs) were absent. No spontaneous or positional nystagmus was observed during the bedside examination with Frenzel goggles. Static posturography, conducted using the SVeP (Standard Vestibology Platform) by Politecnica (Modena, Italy) [15], showed increased sway area (SX, mm^2^) values in all conditions: Eyes Open (EO) = 300; Eyes Closed (EC) = 520; Pad (on foam pads) EO = 350; Pad EC = 580. The patient also reported episodic dizziness without vertigo and persistent bilateral tinnitus in daily life, but not hyperacusis. His Tinnitus Handicap Inventory (THI) score was 60 (range: 0–100), corresponding to Grade 4, which indicates a severe impact on his quality of life due to tinnitus. HR-MRI (high-resolution magnetic resonance imaging) of the brain, performed using a 3 Tesla scanner and incorporating 3D-FLAIR (three-dimensional fluid-attenuated inversion recovery) sequences taken 10 min and 4 h post-gadolinium injection [16], revealed no abnormalities. Similarly, HR-CBCT (high-resolution cone beam computed tomography) of the ear showed negative findings, aside from mild opacification of the mastoid air cells. Given the severity of the SNHL and poor speech discrimination scores, the patient was evaluated for cochlear implantation. However, he declined the procedure due to fear of surgery. As a result, he continued using BTE hearing aids, although the benefit remains limited. Speech discrimination with amplification reaches only 50% in the right ear and 40% in the left, indicating suboptimal auditory rehabilitation. Nevertheless, the patient opted to maintain use of the hearing aids, as they provide partial auditory access sufficient for basic daily communication. He is currently undergoing regular audiological follow-up every six months to monitor disease progression and reassess both his candidacy and willingness for cochlear implantation in the future.

### 2.4. Genetic Tests

A targeted next-generation sequencing (NGS) panel for genes associated with PCD (ACVR2B, CCDC39, CCDC40, CFC1, CRELD1, DNAAF3, DNAH5, DNAH11, DNAI1, DNAI2, DNAL1, FOXH1, GJA1, INVS/HPHP2, KTU/DNAAF2, LEFTY2, LRRC50/DNAAF1, NKX2-5, NODAL, TXNDC3/NME8, ZIC3) returned negative results. However, based on the clinical presentation and ultrastructural ciliary defects, a diagnosis of PCD was confirmed.

To investigate the etiology of the patient’s SNHL, an NGS panel for hereditary deafness genes (ABHD12, ACTG1, ATP6V1B1, BSND, CCDC50, CDH23, CLDN14, CLRN1, COCH, COL11A2, CRYM, DFNA5, DFNB31, DFNB59, DIAPH1, ESPN, ESRRB, EYA1, EYA4, GIPC3, GJB2, GJB3, GJB6, GPR98, GPSM2, GRHL2, GRXCR1, HGF, ILDR1, KCNE1, KCNQ1, KCNQ4, LHFPL5, LOXHD1, LRTOMT, MARVELD2, MIR96, MPZ, MSRB3, MYH14, MYH9, MYO15A, MYO1A, MYO3A, MYO6, MYO7A, NEFL, OTOA, OTOF, PCDH15, PDZD7, PMP22, POU3F4, POU4F3, PRPS1, RDX, SERPINB6, SH3TC2, SLC17A8, SLC26A4 (PDS), SLC26A5, TECTA, TIMM8A, TJP2, TMC1, TMIE, TMPRSS3, TPRN, TRIOBP, USH1C, USH1G, USH2A, WFS1) was performed.

Two heterozygous variants were identified: a missense variant in SLC26A4 (NM_000441.2:c.1234G>T; NP_000432.1:p.Val412Ile) and a nonsense variant in SH3TC2 (NM_024577.4:c.3325C>T; NP_078853.2:p.Arg1109Ter). The SLC26A4 p.Val412Ile variant is considered of uncertain significance (VUS), whereas the SH3TC2 p.Arg1109X variant is classified as pathogenic according to the ACMG/AMP (American College of Medical Genetics and Genomics/Association for Molecular Pathology) classification guidelines [17].

In light of the genetic findings, we proceeded with nerve conduction studies and electromyography to assess for possible peripheral neuropathy; however, the results were normal, indicating no detectable abnormalities in peripheral nerve function.

## 3. Discussion

This case highlights an unusual presentation of PCD with bilateral SNHL in the presence of a pathogenic SH3TC2 mutation. While HL in PCD is typically conductive, some recent studies have reported an increased prevalence of SNHL in PCD patients, ranging from 10.5% to 33% [12,18,19]. The exact mechanism underlying mixed HL or SNHL in PCD remains uncertain, though hypotheses include chronic inflammation leading to fibrosis and calcification of the middle ear, cochlear damage due to recurrent infections, ototoxic effects of antibiotics frequently used in PCD management (e.g., aminoglycosides), and undiagnosed genetic factors [12,19]. PCD is a hereditary ciliary disorder characterized by chronic oto-sino-pulmonary manifestations. In our patient, the diagnosis was confirmed based on the presence of situs inversus, clinical symptoms, and the absence of outer dynein arms in ciliary cross-sections. The absence of mutations in genes associated with PCD is not unexpected, as such mutations are undetected in approximately one-third of PCD cases [20]. Genetic analysis for hereditary HL identified two variants, with only the nonsense variant p.Arg1109X in the SH3TC2 gene classified as pathogenic [17]. The SH3TC2 gene is located on chromosome 5q32 and encodes a protein expressed in Schwann cells of peripheral nerves. Mutations in this gene have been linked to CMT4C, suggesting its role as a Rab11 effector. The p.Arg1109X variant in the SH3TC2 gene is extremely rare in the general population, with a higher frequency in Spanish Gypsies affected by CMT4C [21,22]. Although this patient exhibited mild balance disorders and scoliosis—features seen in CMT4C—he did not display overt peripheral neuropathy. The absence of a second pathogenic SH3TC2 mutation suggests that he does not have full-blown CMT4C—which generally manifests earlier in life—but raises the possibility of subclinical peripheral nervous system involvement, which could plausibly explain his balance difficulties. Given that cranial nerve dysfunction is a hallmark of CMT4C, the presence of SNHL may suggest an underlying neuropathic contribution to his auditory symptoms. Indeed, SH3TC2 mutations are well-recognized for their association with a broad spectrum of HL, ranging from mild to profound, and frequently linked to neuropathic involvement [23]. While the presence of OAEs would have bolstered this hypothesis, their absence may reflect either the severity of the SNHL or cumulative outer hair cell damage from lifelong ototoxic exposures. However, the disproportionate difficulty with speech comprehension relative to pure-tone audiometry findings may support a possible diagnosis of auditory neuropathy spectrum disorder. It remains unclear whether in this patient the SH3TC2 mutation directly contributed to his SNHL or if this finding is coincidental in the context of PCD.

Notably, PCD-related HL is generally conductive, often arising from chronic OME or recurrent AOM. Although maturation of Eustachian tube function with age may lead to hearing improvement [4,24], chronic inflammation and structural middle ear damage can result in long-lasting auditory deficit [25]. The mastoid condensation observed on the CBCT scan of the ear is consistent with secondary changes associated with a history of middle ear inflammation, a common occurrence in these patients [18]. However, some patients experience persistent or progressive SNHL, prompting inquiries into additional mechanisms, including potential genetic predispositions [18,19]. One remarkable case involved three brothers with situs solitus and classical retinitis pigmentosa, who were also diagnosed with PCD and exhibited a slowly progressive bilateral HL [26]. A study by Zawawi et al. investigating otolaryngological manifestations in 47 children with PCD identified one case of severe bilateral mixed HL [27]. Additionally, in our previous study evaluating audiological outcomes in adults with PCD, SNHL was observed in 1 out of 23 patients [28]. The dizziness and postural instability observed in our patient further highlight the need for vestibular evaluations in PCD patients, as both recurrent AOM and sensory cilia dysfunction in the vestibular system may contribute to balance disorders. Childhood exposure to ototoxic antibiotics, which are frequently used in PCD management, could also play a role [15,19]. Kinocilia in cochlear hair cells share molecular pathways with motile cilia, including those involved in ciliogenesis, vesicular trafficking, and planar cell polarity [29,30]. The SH3TC2 gene, which regulates endocytic recycling via Rab11, may affect intracellular trafficking in sensory cells, potentially exacerbating auditory dysfunction when mutated [31].

This study has several limitations. First, while the patient exhibited features suggestive of subclinical neuropathy, no formal neurophysiological studies (e.g., nerve conduction studies or electromyography) confirmed peripheral nerve involvement. Second, in the absence of a second pathogenic SH3TC2 mutation, it remains uncertain whether this variant alone can contribute to auditory dysfunction in heterozygosity. Third, while cochlear damage from chronic infections or ototoxic exposure remains a plausible contributor to the patient’s SNHL, the exact interplay between PCD-related factors and potential genetic predisposition could not be fully elucidated. Finally, the rarity and atypical presentation of this case limit its generalizability, emphasizing the importance of larger cohort studies to clarify the prevalence and clinical significance of SH3TC2 variants in patients with PCD and SNHL. Indeed, it should be recognized that the proposed correlation remains within speculative boundaries, being based primarily on observational findings rather than definitive mechanistic evidence.

Given that SNHL has been reported in up to one-third of PCD patients [19], further research is warranted to investigate the role of ciliary dysfunction in cochlear integrity. Future studies should evaluate whether SH3TC2 mutations in heterozygosity contribute to subclinical neuropathic manifestations, particularly in the auditory and vestibular systems.

## 4. Conclusions

PCD is predominantly associated with upper airway involvement, and chronic OME frequently leads to conductive HL, particularly in childhood. However, in this patient, the presence of SNHL suggests the involvement of additional or alternative etiopathogenic mechanisms, such as ciliary dysfunction or neuropathy. Indeed, the identification of a pathogenic SH3TC2 mutation, commonly associated with CMT4C, raises the possibility of an underlying genetic neuropathy contributing to the patient’s HL and postural instability. Although SH3TC2 mutations in heterozygosity are not known to cause CMT4C, their role in predisposing to subclinical neuropathic manifestations remains uncertain. This case underscores the need for comprehensive audiological assessments and genetic investigations in patients with PCD, particularly when HL is progressive, disproportionately severe, accompanied by poor speech discrimination, or associated with a history of parental consanguinity. Early diagnosis and intervention are crucial in preventing further deterioration of auditory function, ultimately improving quality of life in these patients.

## Data Availability

Data are contained within the article.

## References

[1] Mirra V., Werner C., Santamaria F. (2017). Primary Ciliary Dyskinesia: An Update on Clinical Aspects, Genetics, Diagnosis, and Future Treatment Strategies. Front. Pediatr..

[2] Zariwala M.A., Knowles M.R., Leigh M.W., Adam M.P., Feldman J., Mirzaa G.M., Pagon R.A., Wallace S.E., Amemiya A. (2007). Primary Ciliary Dyskinesia. GeneReviews^®^ [Internet].

[3] Piatti G., Girotto G., Concas M.P., Braga L., Ambrosetti U., Aldè M. (2024). TAS2R38 Genotype Does Not Affect SARS-CoV-2 Infection in Primary Ciliary Dyskinesia. Int. J. Mol. Sci..

[4] Despotes K.A., Zariwala M.A., Davis S.D., Ferkol T.W. (2024). Primary Ciliary Dyskinesia: A Clinical Review. Cells.

[5] Tinoco E.M., Gigante A.R., Ferreira E., Sanches I., Pereira R., Sá R., Monteiro R., Sousa M., Pascoal I. (2023). Primary Ciliary Dyskinesia in a Portuguese Bronchiectasis Outpatient Clinic. Genes.

[6] Alsamri M.T., Alabdouli A., Iram D., Alkalbani A.M., Almarzooqi A.S., Souid A.-K., Vijayan R. (2021). A Study on the Genetics of Primary Ciliary Dyskinesia. J. Clin. Med..

[7] Nagappa M., Sharma S., Taly A.B. (2025). Charcot-Marie-Tooth Disease. StatPearls.

[8] Timmerman V., Strickland A.V., Züchner S. (2014). Genetics of Charcot-Marie-Tooth (CMT) Disease within the Frame of the Human Genome Project Success. Genes.

[9] Stavrou M., Sargiannidou I., Georgiou E., Kagiava A., Kleopa K.A. (2021). Emerging Therapies for Charcot-Marie-Tooth Inherited Neuropathies. Int. J. Mol. Sci..

[10] Cortese A., Wilcox J.E., Polke J.M., Poh R., Skorupinska M., Rossor A.M., Laura M., Tomaselli P.J., Houlden H., Shy M.E. (2020). Targeted next-generation sequencing panels in the diagnosis of Charcot-Marie-Tooth disease. Neurology.

[11] Jerath N.U., Mankodi A., Crawford T.O., Grunseich C., Baloui H., Nnamdi-Emeratom C., Schindler A.B., Heiman-Patterson T., Chrast R., Shy M.E. (2018). Charcot-Marie-Tooth Disease type 4C: Novel mutations, clinical presentations, and diagnostic challenges. Muscle Nerve.

[12] Avgeri C., Sideris G., Moriki D., Douros K., Delides A., Nikolopoulos T. (2025). Bilateral Hearing Loss in Primary Ciliary Dyskinesia: A Study of Conductive and Sensorineural Mechanisms from Pediatric and Adult Cases. J. Int. Adv. Otol..

[13] Sivera R., Cavalle L., Vílchez J.J., Espinós C., Pérez Garrigues H., Sevilla T. (2017). Audiological Findings in Charcot-Marie-Tooth Disease Type 4C. J. Int. Adv. Otol..

[14] Aldè M., Ambrosetti U., Barozzi S., Aldè S. (2025). The Ongoing Challenges of Hearing Loss: Stigma, Socio-Cultural Differences, and Accessibility Barriers. Audiol. Res..

[15] Aldè M., Bosi P., Muck S., Mayr T., Di Mauro P., Berto V., Aleandri B.G., Folino F., Barozzi S., Zanetti D. (2024). Long-Term Impact of Recurrent Acute Otitis Media on Balance and Vestibular Function in Children. Brain Sci..

[16] Aldè M., Ambrosetti U., Piatti G., Romanini C., Filipponi E., Di Berardino F., Zanetti D., Pignataro L., Cantarella G., Barozzi S. (2024). Sudden Sensorineural Hearing Loss in Patients Aged from 15 to 40 Years. J. Clin. Med..

[17] Richards S., Aziz N., Bale S., Bick D., Das S., Gastier-Foster J., Grody W.W., Hegde M., Lyon E., Spector E. (2015). Standards and guidelines for the interpretation of sequence variants: A joint consensus recommendation of the American College of Medical Genetics and Genomics and the Association for Molecular Pathology. Genet. Med..

[18] Alexandru M., de Boissieu P., Benoudiba F., Moustarhfir M., Kim S., Bequignon É., Honoré I., Garcia G., Mitri-Frangieh R., Legendre M. (2022). Otological Manifestations in Adults with Primary Ciliary Dyskinesia: A Controlled Radio-Clinical Study. J. Clin. Med..

[19] Bequignon E., Dupuy L., Zerah-Lancner F., Bassinet L., Honoré I., Legendre M., Devars du Mayne M., Escabasse V., Crestani B., Maître B. (2019). Critical Evaluation of Sinonasal Disease in 64 Adults with Primary Ciliary Dyskinesia. J. Clin. Med..

[20] Shapiro A.J., Zariwala M.A., Ferkol T., Davis S.D., Sagel S.D., Dell S.D., Rosenfeld M., Olivier K.N., Milla C., Daniel S.J. (2016). Diagnosis, monitoring, and treatment of primary ciliary dyskinesia: PCD foundation consensus recommendations based on state of the art review. Pediatr. Pulmonol..

[21] Gooding R., Colomer J., King R., Angelicheva D., Marns L., Parman Y., Chandler D., Bertranpetit J., Kalaydjieva L. (2005). A novel Gypsy founder mutation, p.Arg1109X in the CMT4C gene, causes variable peripheral neuropathy phenotypes. J. Med. Genet..

[22] Claramunt R., Sevilla T., Lupo V., Cuesta A., Millán J.M., Vílchez J.J., Palau F., Espinós C. (2007). The p.R1109X mutation in SH3TC2 gene is predominant in Spanish Gypsies with Charcot-Marie-Tooth disease type 4. Clin. Genet..

[23] Lerat J., Magdelaine C., Lunati A., Dzugan H., Dejoie C., Rego M., Beze Beyrie P., Bieth E., Calvas P., Cintas P. (2019). Implication of the SH3TC2 gene in Charcot-Marie-Tooth disease associated with deafness and/or scoliosis: Illustration with four new pathogenic variants. J. Neurol. Sci..

[24] Andersen T.N., Alanin M.C., von Buchwald C., Nielsen L.H. (2016). A longitudinal evaluation of hearing and ventilation tube insertion in patients with primary ciliary dyskinesia. Int. J. Pediatr. Otorhinolaryngol..

[25] Goutaki M., Lam Y.T., Alexandru M., Anagiotos A., Armengot M., Boon M., Burgess A., Caversaccio N., Crowley S., Dheyauldeen S.A.D. (2023). Characteristics of Otologic Disease Among Patients with Primary Ciliary Dyskinesia. JAMA Otolaryngol. Head Neck Surg..

[26] Krawczyński M.R., Dmeńska H., Witt M. (2004). Apparent X-linked primary ciliary dyskinesia associated with retinitis pigmentosa and a hearing loss. J. Appl. Genet..

[27] Zawawi F., Shapiro A.J., Dell S., Wolter N.E., Marchica C.L., Knowles M.R., Zariwala M.A., Leigh M.W., Smith M., Gajardo P. (2022). Otolaryngology Manifestations of Primary Ciliary Dyskinesia: A Multicenter Study. Otolaryngol. Head Neck Surg..

[28] Piatti G., De Santi M.M., Torretta S., Pignataro L., Soi D., Ambrosetti U. (2017). Cilia and Ear. Ann. Otol. Rhinol. Laryngol..

[29] Jones C., Roper V.C., Foucher I., Qian D., Banizs B., Petit C., Yoder B.K., Chen P. (2008). Ciliary proteins link basal body polarization to planar cell polarity regulation. Nat. Genet..

[30] Ezan J., Lasvaux L., Gezer A., Novakovic A., May-Simera H., Belotti E., Lhoumeau A.C., Birnbaumer L., Beer-Hammer S., Borg J.P. (2013). Primary cilium migration depends on G-protein signalling control of subapical cytoskeleton. Nat. Cell. Biol..

[31] Senderek J., Bergmann C., Stendel C., Kirfel J., Verpoorten N., De Jonghe P., Timmerman V., Chrast R., Verheijen M.H., Lemke G. (2003). Mutations in a gene encoding a novel SH3/TPR domain protein cause autosomal recessive Charcot-Marie-Tooth type 4C neuropathy. Am. J. Hum. Genet..

